# Growth of the airway smooth muscle layer from late gestation to childhood is mediated initially by hypertrophy and subsequently hyperplasia

**DOI:** 10.1111/resp.14240

**Published:** 2022-03-09

**Authors:** Kimberley C. W. Wang, Graham M. Donovan, Sejal Saglani, Thais Mauad, Alan L. James, John G. Elliot, Peter B. Noble

**Affiliations:** ^1^ School of Human Sciences The University of Western Australia Crawley Western Australia Australia; ^2^ Telethon Kids Institute The University of Western Australia Nedlands Western Australia Australia; ^3^ Department of Mathematics University of Auckland Auckland New Zealand; ^4^ National Heart and Lung Institute Imperial College London London UK; ^5^ Department of Pathology University of São Paulo São Paulo Brazil; ^6^ Department of Pulmonary Physiology and Sleep Medicine West Australian Sleep Disorders Research Institute, Sir Charles Gairdner Hospital Nedlands Western Australia Australia; ^7^ Medical School The University of Western Australia Nedlands Western Australia Australia

**Keywords:** airway smooth muscle, asthma, extracellular matrix, hyperplasia, hypertrophy, ontogeny, remodelling

## Abstract

**Background and objective:**

The airway smooth muscle (ASM) layer thickens during development. Identifying the mechanism(s) for normal structural maturation of the ASM reveals pathways susceptible to disease processes. This study characterized thickening of the ASM layer from foetal life to childhood and elucidated the underlying mechanism in terms of hypertrophy, hyperplasia and extracellular matrix (ECM) deposition.

**Methods:**

Airways from post‐mortem cases were examined from seven different age groups: 22–24 weeks gestation, 25–31 weeks gestation, term (37–41 weeks gestation), <0.5 year, 0.5–1 year, 2–5 years and 6–10 years. The ASM layer area (thickness), the number and size of ASM cells and the volume fraction of ECM were assessed by planimetry and stereology.

**Results:**

From late gestation to the first year of life, normalized ASM thickness more than doubled as a result of ASM hypertrophy. Thereafter, until childhood, the ASM layer grew in proportion to airway size, which was mediated by ASM hyperplasia. Hypertrophy and hyperplasia of ASM were accompanied by a proportional change in ECM such that the broad composition of the ASM layer was constant across age groups.

**Conclusion:**

These data suggest that the mechanisms of ASM growth from late gestation to childhood are temporally decoupled, with early hypertrophy and subsequent proliferation. We speculate that the developing airway is highly susceptible to ASM thickening in the first year of life and that the timing of an adverse event will determine structural phenotype.

## Abstract 2

A period of rapid hypertrophic airway smooth muscle growth occurs in the first year of life, representing a critical window for disruption by disease processes and/or opportunity for clinical intervention.

**See related** Editorial

## INTRODUCTION

There is little doubt regarding the adverse consequences of airway smooth muscle (ASM) remodelling in asthma and that this pathology occurs early in the clinical course of disease. Increased thickness of the ASM layer in asthma exaggerates airway narrowing to contractile provocation^1^ and is associated with more severe disease.^2^ Children with asthma already exhibit a greater proportion of ASM in airway biopsies,^3^ and similar expansion of the ASM is observed in pre‐school children who go on to develop asthma at school age,^4^ that is, remodelling seems to precede diagnosis. In adult life, thickness of the ASM layer does not change and is unaffected by the duration of disease.^2^ If we assume a tight coupling between airway structure–function, there is even evidence of airway pathology from the first year of life. Flow impairment and increased airway reactivity in infancy predict asthma in later life.^5^

Much of our understanding regarding the role of ASM in asthma has been advanced through use of airway tissue acquired post‐mortem or after lung resection surgery. Using such methods, we have shown that ASM thickening in asthma is due to both muscle hypertrophy and hyperplasia, and a proportionate increase in extracellular matrix (ECM).^6^ Whether the same structural changes account for normal developmental growth of the ASM layer is not known but is important to establish as disease processes may interfere and/or upregulate mechanisms controlling tissue expansion, thereby contributing to early airway pathology. There is also no reason to assume that muscle hypertrophy and hyperplasia, and ECM deposition within the ASM layer occur during the same periods of development and indeed the specific timing of any disruptive stimulus could contribute to known phenotypes of ASM remodelling. For instance, hypertrophy occurs in both non‐fatal and fatal asthma, whereas hyperplasia is observed in the latter.^6^ On the other hand, fixed airflow limitation seems to be mediated by a disproportionate increase in ECM within the ASM layer.^7^

The purpose of this study was to characterize normal mechanisms of ASM growth in terms of hypertrophy, hyperplasia and/or ECM deposition. Airway tissue from different regions of the lung was examined across seven different age groups, spanning late gestation to childhood. Findings demonstrate that the mechanism of ASM growth is due to early hypertrophy and late hyperplasia, with proportionate expansion of the ECM within the ASM layer.

## METHODS

### Study participants

Tissue is stored in the Airway Disease Biobank at Sir Charles Gairdner Hospital. Airways examined in the present study were sourced from post‐mortem studies of asthma based in Perth (1), London (24), Sydney (13) and São Paulo (13). As shown in Table 1, airways from post‐mortem cases were available from seven a priori classified age groups: 22–24 weeks gestational age (GA; *n* = 8), 25–31 weeks GA (*n* = 8), term (37–41 weeks GA; *n* = 8), <0.5 year old (*n* = 8), 0.5–1 year old (*n* = 8), 2–5 years old (*n* = 8) and 6–10 years old (*n* = 3). Information regarding the history of asthma (and cause of death) was sought from next‐of‐kin, hospital files, coroner files, police reports and the subjects' usual medical practitioners. Reported causes of death varied (see Appendix S1 in the Supporting Information) and include still birth, respiratory distress, infection, tumours and trauma. Two cases of death were undetermined, and one case was not recorded. Subjects from 2 years old onwards had no history of asthma or other lung disease. The proportion of males and females was similar across age groups (chi‐square analysis; *p* = 0.6).

**TABLE 1:** Group characteristics

Group	Age	Sex (M:F)
22–24 weeks GA	23 ± 0.3 weeks GA	4:4
25–31 weeks GA	27 ± 0.7 weeks GA	6:2
Term	40 ± 0.5 weeks GA	4:4
<0.5 year	0.17 ± 0.04 year	4:4
0.5–1 year	0.7 ± 0.06 year	3:5
2–5 years	2.1 ± 0.17 years	2:6
6–10 years	7.5 ± 1.0 years	2:1

*Note*: Values are mean ± SEM.

Abbreviations: F, female; GA, gestational age; M, male.

### Tissue preparation

For subjects up to 5 years of age, a random section of lung tissue was fixed and embedded in paraffin. Tissue preparation and subsequent measurement followed protocols optimized to reduce variability and error.^8^ In the 6–10 years group, airways were sampled according to a systematic design,^9^ and also fixed and embedded in paraffin. From each case, two sections of tissue were cut: a thin section (0.5 μm, Masson's trichrome technique) for estimation of tissue volume fractions (ECM or smooth muscle) within the ASM layer, and a thick section (30 μm, haematoxylin) to estimate ASM cell number and size.

### Airway measurements

The median (interquartile range) number of visually transverse airway sections studied per case was 7 (6–10). The reticular basement membrane perimeter (*P*_bm_, the index of airway size^10^) and area of the ASM layer (ASM_area_)^6^ were measured by planimetry on thick sections. Thickness of the ASM layer was calculated from ASM_area_ divided by *P*_bm_. The same thick section was used to measure numerical density of ASM cells (*N*_V_) using the optical disector approach (∑*Q*/(*a* × *h* × ∑*P*)), where *Q* is the number of cells counted per disector, *a* and *h* are the area and height of the disector, respectively, and *P* is the number of disectors counted per section.^11^ For the very smallest of airways (arbitrarily defined as <0.6 mm *P*_bm_), all ASM cells within the layer were counted and *N*_V_ was calculated as: ∑*Q*/(ASM_area_ × *h*). As the depth of the thick section effectively equates to airway length, the total number of ASM cells per millimetre length of airway (*N*_L_) was calculated as: *N*_L_ = *N*_V_ (cells/mm^3^) × ASM_area_ (mm^2^). An increase in N_L_ has previously been used to reflect hyperplasia in the context of asthma diagnosis.^6^ Mean ASM cell volume (*V*_C_) was calculated as the inverse of *N*_V_, corrected for *V*_VASM_ (see below): *V*_C_ = 1/[*N*_V_ × *V*_VASM_].^6^

To assess volume fractions within the ASM layer, two airways per case were randomly selected. Volume fractions of ASM (*V*_VASM_), ECM (*V*_VECM_) and ‘Other’ (*V*_VOTHER_, the space between tissues, non‐muscle cells and vascular structures) within the ASM layer were estimated by point counting.^6^

### Statistical and data analyses

**FIGURE 1:**
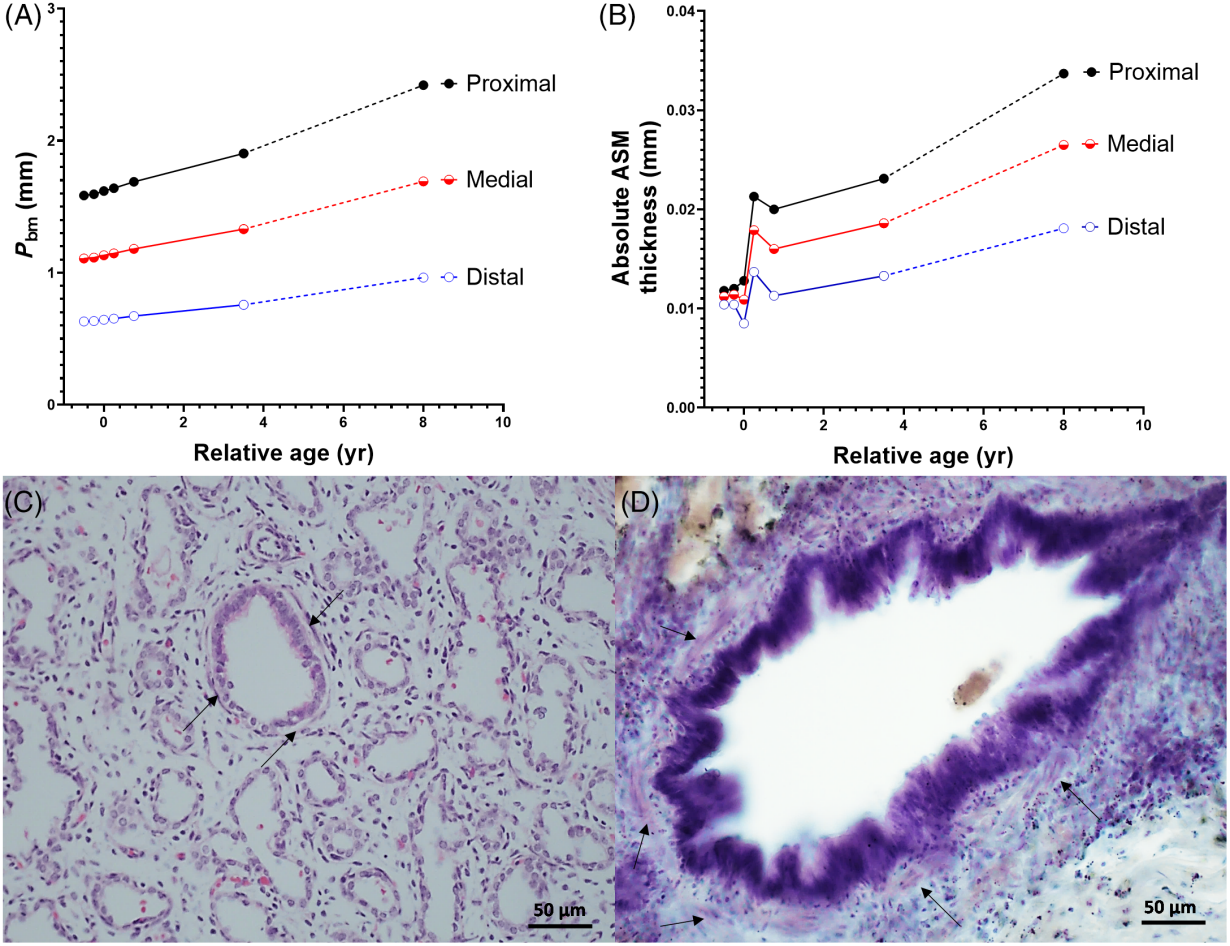
Predictive change in *P*_bm_ (A) and ASM thickness (B) with age and exemplar images (<24 weeks gestational age, C; 6–10 years, D). Arrows indicate ASM. Black circles and line, proximal airways; red circles and line, medial airways; blue circles and line, distal airways. The dashed lines indicate that predictions were based on limited data. ASM, airway smooth muscle; *P*_bm_, perimeter of basement membrane; yr, year(s)

Primary outcomes were ASM thickness (ASM_area_/*P*_bm_), *V*_C_, *N*_L_, *V*_VASM_, *V*_VECM_ and *V*_VOTHER_. For parameters not dependent on airway size (*V*_C_, *V*_VASM_, *V*_VECM_ and *V*_VOTHER_), a case mean was determined by averaging across all measured airways. As ASM thickness and *N*_L_ are dependent on airway size (*P*_bm_), which varies with anatomical location and maturation, we used a method based on Pascoe et al.^12^ to generate a representative and scaled airway for each case across all age groups: (1) ASM thickness and *N*_L_ were first shown to follow a power law = *c* × (*P*_bm_)^*b*^, where *b* describes how the parameter varies with *P*_bm_ and *c* the value at size *P*_bm_ = 1 mm (referred to as ‘normalized’ ASM thickness, *N*_L_ or WAt), and (2) to estimate absolute changes in ASM thickness and *N*_L_, *P*_bm_ growth curves (Figure 1A) were established from computed tomography (CT)‐derived tracheal lumen dimensions,^13^, ^14^ assuming that intraparenchymal airways grow in proportion to the trachea. Growth curves (changes in *P*_bm_ from late gestation to childhood) were generated for an arbitrary distal (*P*_bm_ = 0.63 mm), medial (*P*_bm_ = 1.11 mm) and proximal airway (*P*_bm_ = 1.58 mm) at 22–24 weeks GA to model changes across different airway sizes. Absolute ASM thickness and *N*_L_ could then be calculated from the normalized data (see ‘1’ above), multiplied by *P*_bm_.

Based on data distribution, parameters were analysed using one‐way ANOVA (Holm Sidak's post hoc test) or Kruskal–Wallis one‐way ANOVA on ranks (Dunn's post hoc test) using SigmaPlot (version 13, Chicago, IL, USA). Prism (version 7, San Diego, CA, USA) was used for graphical analyses. Post hoc comparisons were compared with the term group, an appropriate reference for examining in utero and/or postnatal changes. Due to the lower sample size, the 6–10 age group was not included in any categorical statistical analysis. *p* < 0.05 was defined as statistical significance.

## RESULTS

**FIGURE 2:**
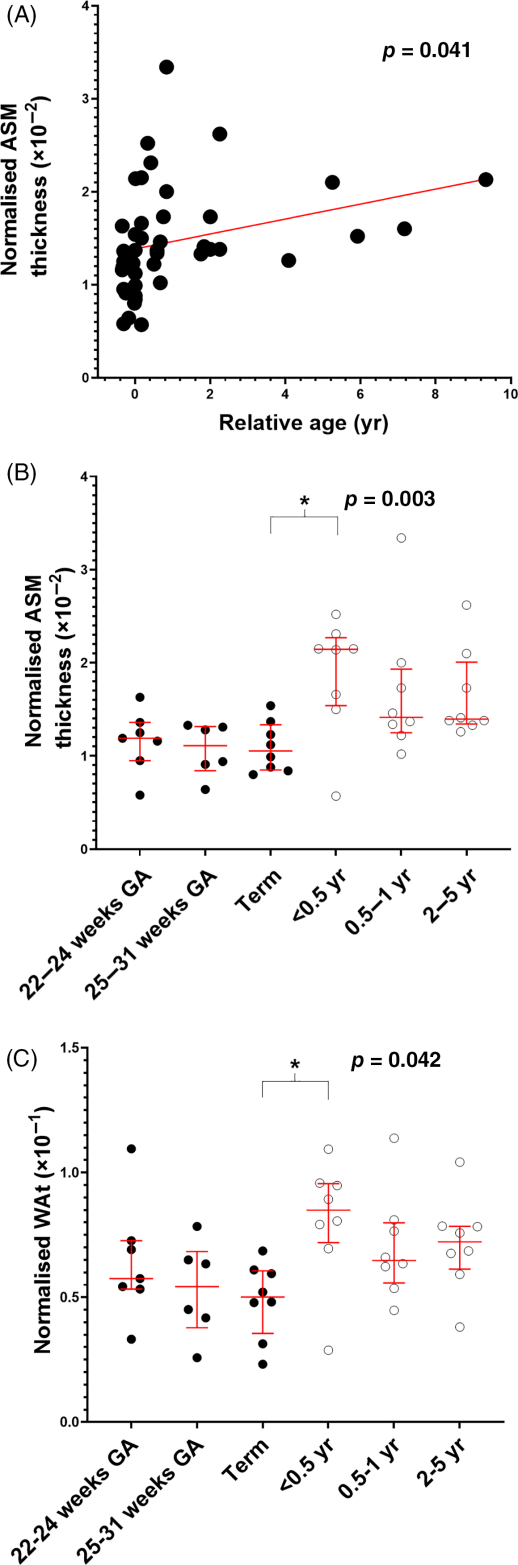
Normalized ASM thickness plotted against continuous age (A) or presented in stratified age groups (B). Normalized total wall thickness presented in stratified age groups (C). Data are median (interquartile range). ASM, airway smooth muscle; GA, gestational age; WAt, total wall thickness; yr, year(s). *Significantly different from term (*p* < 0.05)

Figure 1B indicates predictive ASM growth from late gestation to childhood, which is supported by exemplar images (Figure 1C,D) chosen so that *P*_bm_ as a proportion of the trachea was matched across groups. Normalized ASM thickness was positively correlated with age (*p* = 0.041, *r* = 0.297; Figure 2A). When comparing the a priori classified age groups, much of the growth occurred up until <0.5 year age (*p* = 0.003; Figure 2B). Similar changes were observed in total wall thickness (*p* = 0.042; Figure 2C).

**FIGURE 3:**
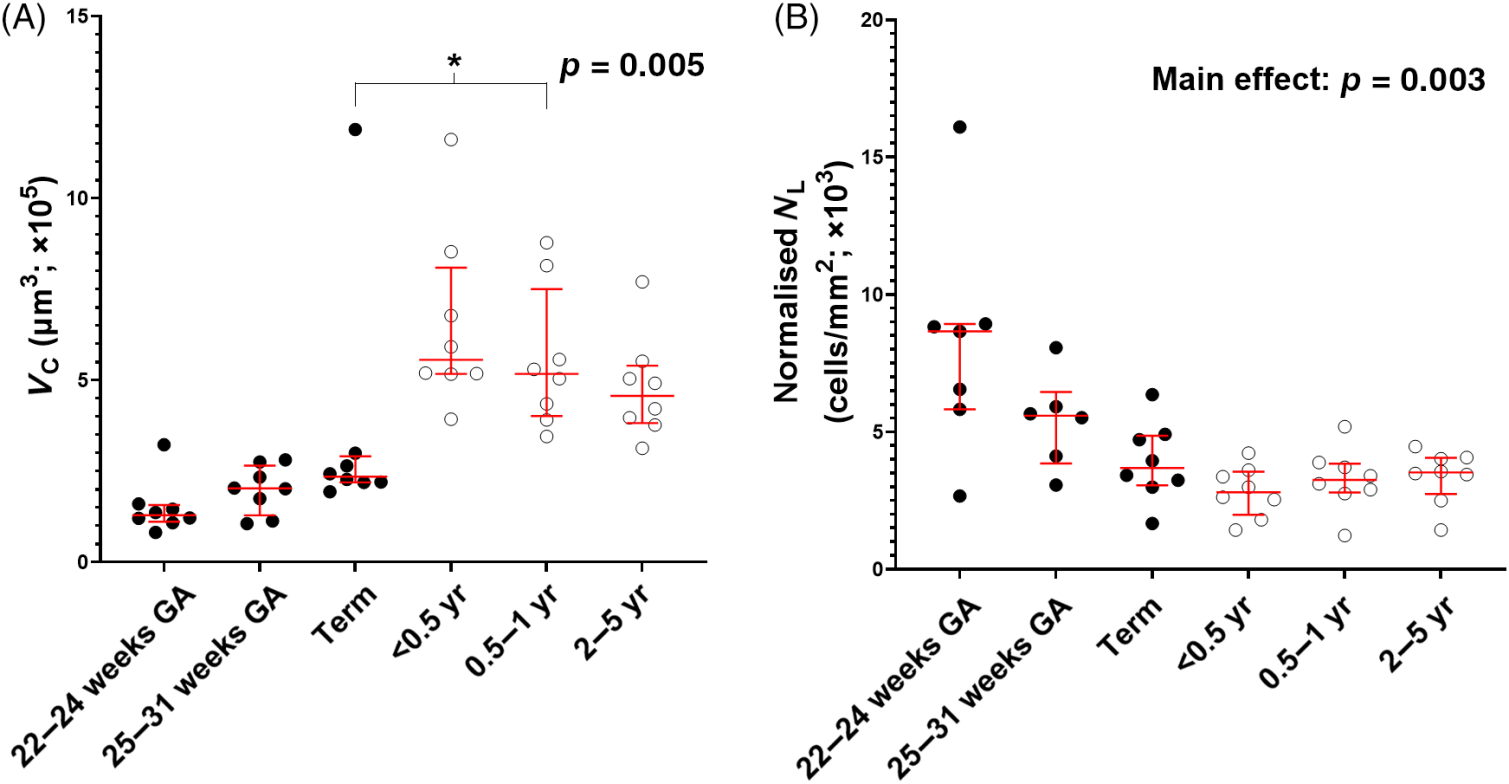
The ASM cell volume (A) and *N*_L_ (B) presented in stratified age groups. Data are median (interquartile range). ASM, airway smooth muscle; GA, gestational age; *N*_L_, total number of ASM cells per millimetre length of airway; *V*_C_, mean ASM cell volume; yr, year(s). *Significantly different from term (*p* < 0.05). ‘Main effect’ indicates an overall effect of age on N_L_; there were no differences after post hoc analysis

Growth of the ASM layer can initially be accounted for by hypertrophy (*p* < 0.001; Figure 3A). The *V*_C_ at 0.5–1 year was greater than term (*p* = 0.005). Changes in *V*_C_ likely caused a reduction in normalized *N*_L_ (*p* = 0.003; Figure 3B), that is, an increase in ASM cell size naturally reduces the number of cells that can be accommodated along a given mm length of airway (i.e., *N*_L_). There was no increase in *V*_C_ after 0.5–1 year of age.

The *V*_VASM_ (*p* = 0.158) and *V*_VECM_ (*p* = 0.150) were not significantly different from pre‐ to postnatal life. Given that ASM thickness increases with age, constant volume fractions support a proportionate increase in the ECM and muscle within the ASM layer during maturation (Table 2). There was an overall main effect of age on *V*_VOTHER_ (*p* = 0.007; Table 2), but no differences between groups after post hoc analysis.

**TABLE 2:** Proportion of *V*_VASM_, *V*_VECM_ and *V*_VOTHER_ within the ASM layer

Group	*V*_VASM_	*V*_VECM_	*V*_VOTHER_ ^a^
22–24 weeks GA	0.82 ± 0.08	0.11 ± 0.11	0.08 ± 0.10
25–31 weeks GA	0.84 ± 0.14	0.10 ± 0.07	0.07 ± 0.12
Term	0.83 ± 0.11	0.12 ± 0.08	0.06 ± 0.10
<0.5 year	0.84 ± 0.19	0.10 ± 0.11	0.07 ± 0.10
0.5–1 year	0.84 ± 0.19	0.08 ± 0.11	0.08 ± 0.12
2–5 years	0.87 ± 0.13	0.11 ± 0.08	0.02 ± 0.05

*Note*: Values are mean ± SEM.

Abbreviations: ASM, airway smooth muscle; GA, gestational age; *V*_VASM_, volume fraction of ASM; *V*_VECM_, volume fraction of extracellular matrix; *V*_VOTHER_, volume fraction of other.

^a^ Significant main effect of age on *V*_VOTHER_ (*p* < 0.05). There was no difference after post hoc analysis.

**FIGURE 4:**
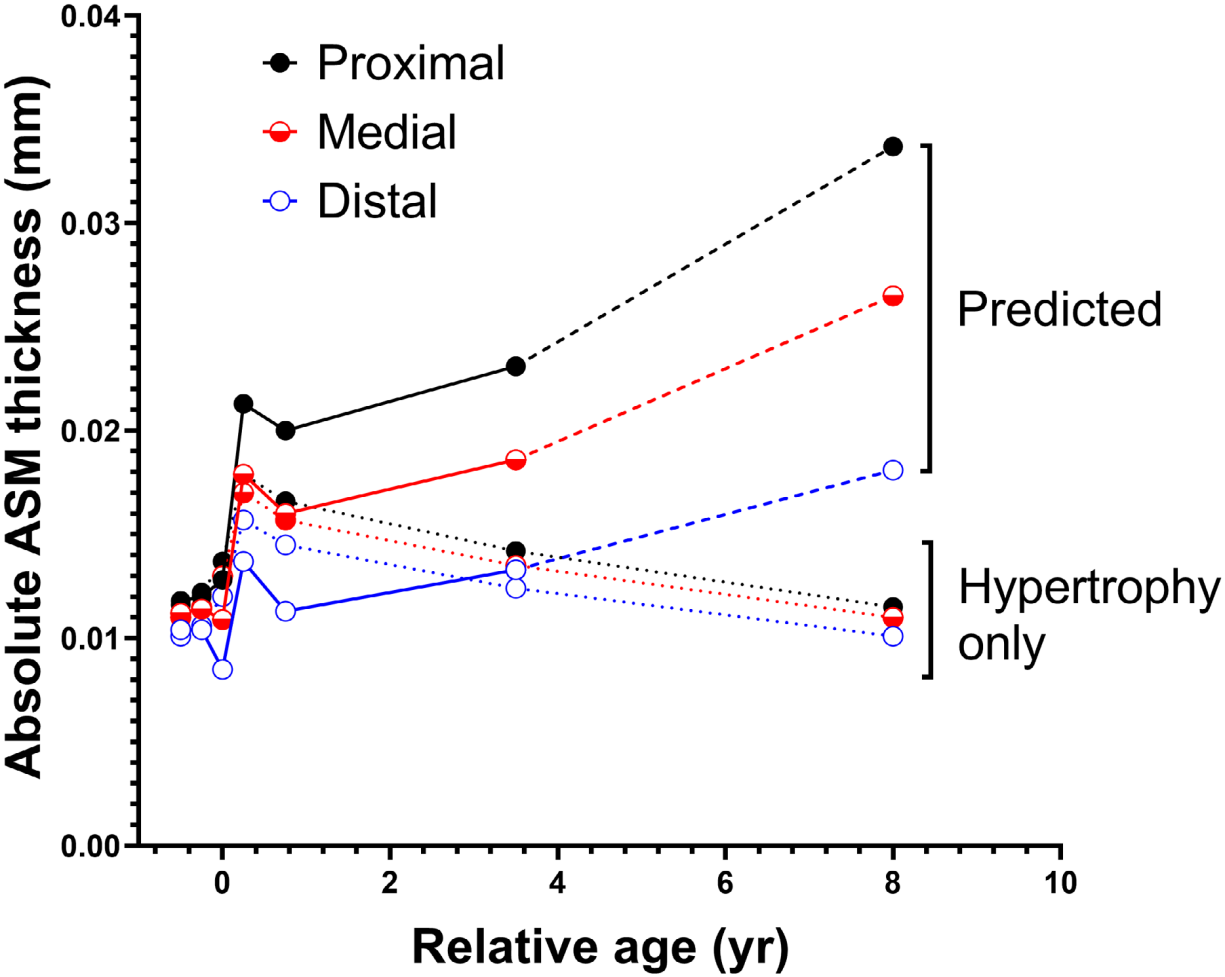
Modelled effects of ASM thickening occurring solely due to isotropic growth of ASM cells without hyperplasia (hypertrophy only; dotted lines) compared with predicted ASM growth (outputs are the same as those presented in Figure 1B). Hypertrophy cannot account for ASM growth beyond 0.5 year. Black circles and lines, proximal airways; red circles and lines, medial airways; blue circles and lines, distal airways. ASM, airway smooth muscle; yr, year(s)

As indicated above, ASM hypertrophy directly impacts the measurement of *N*_L_ which we used to assess hyperplasia. To isolate hyperplasia opposed to hypertrophy (assuming a proportionate increase in ECM as the muscle components expand), we modelled the effects of ASM thickening occurring solely due to hypertrophy (Figure 4). Hypertrophic growth of the ASM layer (dotted lines) was compared with the empirical data (solid/dashed lines). Results indicate that while ASM growth is entirely explained by ASM hypertrophy up and until 0.5 year of age, thereafter, it does not account for ASM thickening, and hyperplasia must be occurring until at least early childhood.

## DISCUSSION

Age‐dependent changes in airway and lung function are well established^15^ and naturally impact the onset and severity of respiratory disease. This study concerned normal growth of the ASM layer, a structure that is prominent in asthma due to its increased thickness, promoting exaggerated constriction of the airway lumen.^1^ Through morphological and stereological examination of human airway tissue, we show that the thickness of the ASM layer increases from late gestation to early childhood, in general agreement with previous findings,^16^ but importantly is initially due to hypertrophy and subsequently hyperplasia. Most notably, during the first year of life, there is rapid and disproportionate ASM growth, marking a period for disease susceptibility and potentially therapeutic intervention.

Morphometric indices of the airway wall are dependent on both airway size and anatomical location (e.g., segmental or sub‐segmental bronchus). In adult subjects, the conventional approach is to match airways based on *P*_bm_, an accepted marker of airway size.^10^ In the context of an ontogeny study, it is perhaps more appropriate to match based on anatomical location, as *P*_bm_ will increase as the airway grows. Random sampling of airways (for most cases) meant that anatomical matching was not possible. Our approach was to establish a single representative airway for each human case based on the relationship between *P*_bm_ and ASM thickness as described by a power law. This regression analysis enabled us to scale each representative airway to a fixed arbitrary size of 1 mm *P*_bm_ and effectively remove the complication of airway size (referred to as normalized ASM thickness). The final step in the analysis was to estimate changes in intra‐parenchymal airway size from late gestation to childhood through interpolation and extrapolation of the available CT‐derived tracheal dimensions.^13^, ^14^ These studies also support proportional growth across generations (our inherent assumption), at least for conducting sized airways.

Results demonstrate two clear phases of ASM growth. Immediately after birth for ~1 year, there is disproportionate thickening of the ASM layer (and also total airway wall) relative to airway size (*P*_bm_), reflected by an increase in normalized ASM thickness, that is, the ASM layer grows more rapidly than the airway lumen dimension. Growth of the ASM layer during the first phase is entirely explained by hypertrophic growth (increased cell volume), as modelled in Figure 4. Under these circumstances, the parameter *N*_L_ becomes less informative and insensitive to hyperplasia as the number of cells that can be contained within a given length of airway is reduced as the volume of each cell unit expands, and hence *N*_L_ tends to fall with age. The second phase of ASM growth is marked by proportionate ASM growth that occurs without any further change in ASM cell volume, and therefore must be due to hyperplasia (assuming no shift in the proportion of ECM; see below). The increased number of ASM cells will be deposited radially as the circumference of the airway increases with age, and also transversely so that the normalized thickness of ASM remains constant.

When assessing expansion of the ASM layer, either in the context of normal or abnormal growth, changes in ECM opposed to ASM tissue require consideration. It is feasible that an increase in ASM thickness occurs through expansion of matrix components rather than contractile elements, as has been observed in patients with fixed airflow limitation.^7^ Volume fractions of ASM and ECM are estimated by use of ultrathin sections (0.5 μm), preventing overlap with tissue viewed in a different plane. Expansion of ECM in preference to ASM would be reflected by an increase and decrease in *V*_VECM_ and *V*_VASM_, respectively. The *V*_VASM_ and *V*_VECM_ were however constant over the age range, with only a small deviation in *V*_VOTHER_ (not significant after post hoc analysis), the parameter most likely to be impacted by histological artefact (i.e., spaces between tissue). Therefore, the composition of the ASM layer was not affected by age, suggesting that ASM (contractile elements) and ECM increase concomitantly and proportionally with age.

Structural modelling (as opposed to pathological remodelling) of the ASM layer with maturation is expected to alter ASM force production and airway narrowing. Longitudinal data show that in children without asthma, airway responsiveness to contractile stimuli decreases from infancy to age 11 years.^17^ Similarly, in other species, airway responsiveness is increased in the immature compared with the mature animal.^18^, ^19^ On the surface, an increase in the thickness of the ASM layer with age is discordant with a decrease in airway responsiveness, although the structure–function relationship is modified by numerous age‐dependent factors including subject and airway size,^20^ contractility (force per cross‐sectional area) of the ASM layer,^21^ airway–parenchymal tethering,^22^ bronchodilatory response to breathing stresses^23^ and mechanical loads opposing bronchoconstriction.^24^, ^25^ In particular, while there is a directly proportional relationship between ASM thickness and tension, according to the Law of Laplace, for a given increase in ASM tension, there will be greater collapse pressure in a smaller immature airway compared with a large mature airway.

It is our assertion that understanding the structural mechanism of normal ASM growth provides insight into the pathophysiology of airway remodelling in respiratory disease. Thickness of the ASM layer is increased in asthma,^2^ sudden infant death syndrome (SIDS)^26^ and chronic obstructive pulmonary disease.^27^ In asthma, remodelling of the ASM layer is observed in early life^3^ which implicates maturational time points as the critical window for disruption by disease processes. Appreciating the central role of ASM remodelling in increasing airway narrowing capacity,^1^ rapid and disproportionate growth of the ASM layer in the first year of life will likely increase the probability of an asthma diagnosis. This speculation is supported circumstantially by the association between increased airway responsiveness in the first year of life and subsequent asthma development.^5^

Access to rare human airway tissue is difficult and there is a scientific compromise, in particular the unknown effects of various uncontrolled extraneous factors such as cause of death and relative maternal health. Reported causes of death were varied and incomplete, which at the very least would have increased the variability within and between age groups. Infants dying from SIDS have increased ASM compared with non‐SIDS infants^26^ and were therefore not included in this study. There is also a compelling argument that maternal health affects foetal lung growth and increases the susceptibility to airway disease.^28^, ^29^, ^30^ Adverse foetal lung growth could certainly extend to the ASM layer. For example, maternal hypoxia in mice restricts foetal growth and increases ASM thickness.^31^ The above confounders are acknowledged, albeit there is no reason to suggest that random error affects the trajectory and mechanism of ASM growth, and therefore the conclusions drawn from the present study.

In summary, this study described several phases of ASM development that account for the increase in ASM thickness from late gestation to childhood. Specifically, we observed early ASM hypertrophy and subsequent hyperplasia, and an accompanying expansion of the ECM, such that the overall composition of the ASM layer remained unchanged. Characterizing these structural mechanisms governing normal ASM growth helps advance our understanding on the impact of perinatal influences on airway development and the onset of variable or fixed airflow obstruction in childhood and later life.

## CONFLICT OF INTEREST

None declared.

## AUTHOR CONTRIBUTION

**Kimberley C. W. Wang:** Data curation (lead); formal analysis (lead); funding acquisition (lead); investigation (lead); methodology (equal); project administration (lead); resources (lead); software (lead); supervision (lead); validation (lead); visualization (lead); writing – original draft (lead); writing – review and editing (lead). **Graham M. Donovan:** Data curation (equal); formal analysis (equal); methodology (equal); software (equal); visualization (equal); writing – review and editing (supporting). **Sejal Saglani:** Data curation (supporting); investigation (supporting); writing – review and editing (supporting). **Thais Mauad:** Data curation (supporting); investigation (supporting); writing – review and editing (supporting). **Alan L. James:** Conceptualization (supporting); data curation (supporting); funding acquisition (supporting); investigation (supporting); methodology (supporting); software (supporting); supervision (supporting); writing – original draft (equal); writing – review and editing (equal). **John G. Elliot:** Conceptualization (supporting); data curation (supporting); formal analysis (supporting); funding acquisition (supporting); methodology (supporting); software (supporting); writing – review and editing (supporting). **Peter B. Noble:** Conceptualization (equal); formal analysis (equal); funding acquisition (lead); investigation (supporting); methodology (supporting); resources (supporting); software (supporting); supervision (equal); validation (equal); visualization (equal); writing – original draft (equal); writing – review and editing (equal).

## HUMAN ETHICS APPROVAL DECLARATION

This study was approved by the institutional ethics committees of the participating centres and from the Sir Charles Gairdner Hospital Human Research Ethics Committee (HREC 2015‐53).

## Supporting information


**Supporting information**.

## Data Availability

The data that support the findings of this study are available on request from the corresponding author. The data are not publicly available due to privacy or ethical restrictions.

## References

[1] Noble PB , Jones RL , Cairncross A , Elliot JG , Mitchell HW , James AL , et al. Airway narrowing and bronchodilation to deep inspiration in bronchial segments from subjects with and without reported asthma. J Appl Physiol (1985). 2013;114:1460–71. 23493364 10.1152/japplphysiol.01489.2012

[2] James AL , Bai TR , Mauad T , Abramson MJ , Dolhnikoff M , McKay KO , et al. Airway smooth muscle thickness in asthma is related to severity but not duration of asthma. Eur Respir J. 2009;34:1040–5. 19282340 10.1183/09031936.00181608

[3] Regamey N , Ochs M , Hilliard TN , Mühlfeld C , Cornish N , Fleming L , et al. Increased airway smooth muscle mass in children with asthma, cystic fibrosis, and non‐cystic fibrosis bronchiectasis. Am J Respir Crit Care Med. 2008;177:837–43. 18218992 10.1164/rccm.200707-977OC

[4] O'Reilly R , Ullmann N , Irving S , Bossley CJ , Sonnappa S , Zhu J , et al. Increased airway smooth muscle in preschool wheezers who have asthma at school age. J Allergy Clin Immunol. 2013;131:1024–32. 23069488 10.1016/j.jaci.2012.08.044

[5] Owens L , Laing IA , Zhang G , Le Souëf PN . Infant lung function predicts asthma persistence and remission in young adults. Respirology. 2017;22:289–94. 27637998 10.1111/resp.12901

[6] James AL , Elliot JG , Jones RL , Carroll ML , Mauad T , Bai TR , et al. Airway smooth muscle hypertrophy and hyperplasia in asthma. Am J Respir Crit Care Med. 2012;185:1058–64. 22403800 10.1164/rccm.201110-1849OC

[7] Jones RL , Noble PB , Elliot JG , Mitchell HW , McFawn PK , Hogg JC , et al. Airflow obstruction is associated with increased smooth muscle extracellular matrix. Eur Respir J. 2016;47:1855–7. 26989102 10.1183/13993003.01709-2015

[8] Jones RL , Elliot JG , James AL . Estimating airway smooth muscle cell volume and number in airway sections. Sources of variability. Am J Respir Cell Mol Biol. 2014;50:246–52. 24007332 10.1165/rcmb.2013-0029OC

[9] Mauad T , Silva LF , Santos MA , Grinberg L , Bernardi FD , Martins MA , et al. Abnormal alveolar attachments with decreased elastic fiber content in distal lung in fatal asthma. Am J Respir Crit Care Med. 2004;170:857–62. 15151920 10.1164/rccm.200403-305OC

[10] James AL , Hogg JC , Dunn LA , Paré PD . The use of the internal perimeter to compare airway size and to calculate smooth muscle shortening. Am Rev Respir Dis. 1988;138:136–9. 3202392 10.1164/ajrccm/138.1.136

[11] Sterio DC . The unbiased estimation of number and sizes of arbitrary particles using the disector. J Microsc. 1984;134:127–36. 6737468 10.1111/j.1365-2818.1984.tb02501.x

[12] Pascoe CD , Seow CY , Hackett TL , Paré PD , Donovan GM . Heterogeneity of airway dimensions in humans: a critical determinant of lung function in asthmatics and non‐asthmatics. Am J Physiol Lung Cell Mol Physiol. 2017;312:L425–L431. 28062484 10.1152/ajplung.00421.2016

[13] Rao L , Tiller C , Coates C , Kimmel R , Applegate KE , Granroth‐Cook J , et al. Lung growth in infants and toddlers assessed by multi‐slice computed tomography. Acad Radiol. 2010;17:1128–35. 20542449 10.1016/j.acra.2010.04.012 PMC2918706

[14] Kuo W , Ciet P , Andrinopoulou ER , Chen Y , Pullens B , Garcia‐Peña P , et al. Reference values for central airway dimensions on CT images of children and adolescents. AJR Am J Roentgenol. 2018;210:423–30. 29261353 10.2214/AJR.17.18597

[15] Sharma G , Goodwin J . Effect of aging on respiratory system physiology and immunology. Clin Interv Aging. 2006;1:253–60. 18046878 10.2147/ciia.2006.1.3.253 PMC2695176

[16] Hislop AA , Haworth SG . Airway size and structure in the normal fetal and infant lung and the effect of premature delivery and artificial ventilation. Am Rev Respir Dis. 1989;140:1717–26. 2604298 10.1164/ajrccm/140.6.1717

[17] Cox DW , Mullane D , Zhang GC , Turner SW , Hayden CM , Goldblatt J , et al. Longitudinal assessment of airway responsiveness from 1 month to 18 years in the PIAF birth cohort. Eur Respir J. 2015;46:1654–61. 26493795 10.1183/13993003.00397-2015

[18] Shen X , Bhargava V , Wodicka GR , Doerschuk CM , Gunst SJ , Tepper RS . Greater airway narrowing in immature than in mature rabbits during methacholine challenge. J Appl Physiol (1985). 1996;81:2637–43. 9018516 10.1152/jappl.1996.81.6.2637

[19] Mitchell HW , McFawn PK , Sparrow MP . Increased narrowing of bronchial segments from immature pigs. Eur Respir J. 1992;5:207–12. 1559585

[20] Le Souëf PN , Sears MR , Sherrill D . The effect of size and age of subject on airway responsiveness in children. Am J Respir Crit Care Med. 1995;152:576–9. 7633710 10.1164/ajrccm.152.2.7633710

[21] Sparrow MP , Mitchell HW . Contraction of smooth muscle of pig airway tissues from before birth to maturity. J Appl Physiol (1985). 1990;68:468–77. 2318758 10.1152/jappl.1990.68.2.468

[22] Mansell AL , McAteer AL , Oldmixon EH . Mechanical dissociation of bronchi from parenchyma in the immature piglet lung. J Appl Physiol (1985). 2000;89:228–34. 10904056 10.1152/jappl.2000.89.1.228

[23] Weist A , Williams T , Kisling J , Clem C , Tepper RS . Volume history and effect on airway reactivity in infants and adults. J Appl Physiol (1985). 2002;93:1069–74. 12183504 10.1152/japplphysiol.00986.2001

[24] McFawn PK , Mitchell HW . Bronchial compliance and wall structure during development of the immature human and pig lung. Eur Respir J. 1997;10:27–34. 9032487 10.1183/09031936.97.10010027

[25] Ramchandani R , Shen X , Gunst SJ , Tepper RS . Comparison of elastic properties and contractile responses of isolated airway segments from mature and immature rabbits. J Appl Physiol (1985). 2003;95:265–71. 12794098 10.1152/japplphysiol.00362.2002

[26] Elliot J , Vullermin P , Carroll N , James A , Robinson P . Increased airway smooth muscle in sudden infant death syndrome. Am J Respir Crit Care Med. 1999;160:313–6. 10390417 10.1164/ajrccm.160.1.9802024

[27] Hogg JC . Pathophysiology of airflow limitation in chronic obstructive pulmonary disease. Lancet. 2004;364:709–21. 15325838 10.1016/S0140-6736(04)16900-6

[28] Piedimonte G , Harford TJ . Effects of maternal‐fetal transmission of viruses and other environmental agents on lung development. Pediatr Res. 2020;87:420–6. 31698410 10.1038/s41390-019-0657-4 PMC6962526

[29] Wang KCW , James AL , Noble PB . Fetal growth restriction and asthma; is the damage done? Physiology. 2021;36:256–66. 34159809 10.1152/physiol.00042.2020

[30] Stocks J , Hislop A , Sonnappa S . Early lung development: lifelong effect on respiratory health and disease. Lancet Respir Med. 2013;1:728–42. 24429276 10.1016/S2213-2600(13)70118-8

[31] Wang KCW , Noble PB . Foetal growth restriction and asthma: airway smooth muscle thickness rather than just lung size? Respirology. 2020;25:889–91. 32488950 10.1111/resp.13851

