# Supplementary material for: Growth of the airway smooth muscle layer from late gestation to childhood is mediated initially by hypertrophy and subsequently hyperplasia
Source: Respirology. 2022 Mar 9;27(7):493–500. doi: 10.1111/resp.14240 (PMC9545757; doi:10.1111/resp.14240)
Supplement: Supplementary file 1 — Supporting information. [file RESP-27-493-s001.docx]

**SUPPORTING INFORMATION**

**Growth of the airway smooth muscle layer from late gestation to childhood is mediated initially by hypertrophy and subsequently hyperplasia**

**Kimberley C. W. Wang^1,2^, Graham M. Donovan^3^, Sejal Saglani^4^, Thais Mauad^5^, Alan L. James^6,7^, John G. Elliot^1,6^ and Peter B. Noble^1^**

^1^ School of Human Sciences, The University of Western Australia, Crawley, Western Australia, Australia

^2^ Telethon Kids Institute, The University of Western Australia, Nedlands, Western Australia, Australia

^3^ Department of Mathematics, University of Auckland, Auckland, New Zealand

^4^ Respiratory Paediatrics, Imperial College London, London, United Kingdom

^5^ Department of Pathology, University of São Paulo, São Paulo, Brazil

^6^ Department of Pulmonary Physiology and Sleep Medicine, West Australian Sleep Disorders Research Institute, Sir Charles Gairdner Hospital, Nedlands, Western Australia, Australia

^7^ Medical School, The University of Western Australia, Nedlands, Western Australia, Australia

**Appendix S1-Causes of death**

Reported causes of death are listed verbatim from next-of-kin, hospital files, coroner files, police reports and the subjects’ usual medical practitioners: staphylococcus aureus bacteraemia infection, no respiratory effort at birth, early neonatal death, respiratory distress syndrome, amniocentesis, type 1 thanatophoric dwarfism, still birth, asphyxia, intracranial haemorrhage, necrotising enterocolitis, septicaemia, intraventricular haemorrhage, meconium aspiration, brain tumour, meningitis, endocarditis, cerebral disease, leukemia, medium chain acyl-coA dehydrogenase deficiency, bed sharing accident, thymoma, hepatitis, lymphoma, neuroblastoma, head injury, meningococcal sepsis, multiple injuries due to car accident, compression of the neck, fractured skull and immersion. Two cases of death were undetermined, and one case was not recorded.
